# Unraveling MYC’s Role in Orchestrating Tumor Intrinsic and Tumor Microenvironment Interactions Driving Tumorigenesis and Drug Resistance

**DOI:** 10.3390/pathophysiology30030031

**Published:** 2023-09-11

**Authors:** Zinab O. Doha, Rosalie C. Sears

**Affiliations:** 1 Department of Molecular and Medical Genetics, Oregon Health & Science University, Portland, OR 97239, USA; dohaz@ohsu.edu; 2 Department of Medical Laboratories Technology, Taibah University, Al-Madinah 42353, Saudi Arabia; 3 Brenden-Colson Center for Pancreatic Care, Oregon Health & Science University, Portland, OR 97201, USA; 4 Knight Cancer Institute, Oregon Health & Science University, Portland, OR 97201, USA

**Keywords:** MYC, tumorigenesis, therapeutic resistance, replication stress, DNA repair, immune evasion

## Abstract

The transcription factor MYC plays a pivotal role in regulating various cellular processes and has been implicated in tumorigenesis across multiple cancer types. MYC has emerged as a master regulator governing tumor intrinsic and tumor microenvironment interactions, supporting tumor progression and driving drug resistance. This review paper aims to provide an overview and discussion of the intricate mechanisms through which MYC influences tumorigenesis and therapeutic resistance in cancer. We delve into the signaling pathways and molecular networks orchestrated by MYC in the context of tumor intrinsic characteristics, such as proliferation, replication stress and DNA repair. Furthermore, we explore the impact of MYC on the tumor microenvironment, including immune evasion, angiogenesis and cancer-associated fibroblast remodeling. Understanding MYC’s multifaceted role in driving drug resistance and tumor progression is crucial for developing targeted therapies and combination treatments that may effectively combat this devastating disease. Through an analysis of the current literature, this review’s goal is to shed light on the complexities of MYC-driven oncogenesis and its potential as a promising therapeutic target.

## 1. Objectives

This review paper has two primary objectives: firstly, to provide a comprehensive overview of how MYC influences tumorigenesis and therapeutic resistance, dissecting its role in cell proliferation, survival, DNA repair, immune evasion, angiogenesis and fibroblast remodeling. Secondly, this review aims to highlight MYC’s potential as a therapeutic target for innovative cancer treatment approaches by exploring strategies such as inhibiting MYC expression, destabilizing its protein, disrupting MYC/MAX dimerization and combining MYC inhibitors with DNA-damaging agents.

In summary, this review seeks to comprehensively explore MYC’s intricate role in tumorigenesis and therapeutic resistance and to highlight the promise of MYC as a therapeutic target. By addressing these objectives, we aim to contribute to the growing body of knowledge surrounding MYC-driven oncogenesis and its implications for cancer treatment.

## 2. The Physiological Function of MYC

The *MYC* gene encodes a multifunctional nuclear phosphoprotein that controls a variety of cellular functions. MYC proteins largely function as an essential global transcription factor, regulating genes involved in several different cellular processes, including cell growth, cell cycles, differentiation, apoptosis, angiogenesis, metabolism, DNA repair, protein translation, mitochondrial biogenesis, immune response and stem cell formation [1,2]. Because of its ability to regulate widespread gene expression, MYC expression is tightly controlled in normal cells. MYC activation in normal cells is prevented from causing tumorigenesis through multiple genetic and epigenetically controlled checkpoint mechanisms, including proliferative arrest, apoptosis and cellular senescence [3].

The *MYC* gene family consists of three members, *c-MYC*, *L-MYC* and *N-MYC*, with c-MYC being the most widely expressed member and all containing essentially the same conserved regions that are functionally important [2,4,5]. All contain three domain-type structures, an N-terminal region containing the transactivation domain with conserved regions known as MYC boxes (MB); a central region, also containing conserved MBs, implicated in nuclear localization as well as stability control; and a C-terminal region involved in binding to DNA and comprising the basic helix–loop–helix leucine zipper (bHLHZ) domain [6]. MYC dimerizes with its partner MAX through bHLHZ domains resulting in a stable DNA-binding heterodimer, which is essential for MYC to regulate gene transcription [2,6]. The primary mechanism by which MYC regulates gene expression is through the binding of MYC/MAX heterodimers to E-Box sequences in the regulatory regions of target genes [2,6].

## 3. MYC Is Often Activated in Human Cancers

MYC alterations have been reported to occur in approximately 70% of human malignancies [2,7]. MYC can be genetically activated directly through genomic amplification, chromosomal translocation, retroviral integration, the activation of super enhancers and mutations [2,8,9,10]. Additionally, MYC can be activated through downstream growth signaling from other oncogenes, including RAS, SRC and NOTCH [9,11], or the inactivation of tumor suppressor genes, such as Adenomatous polyposis coli (*APC*), Phosphatase and tensin homolog (*PTEN*) and Protein phosphatase 2A *PP2A* [12,13,14,15], leading to increased *MYC* gene expression, translation and/or protein stability. 

It has been demonstrated that even relatively small constitutive changes in the *MYC* expression level >2-fold -fold relative to normal) have biological consequences and impact tumorigenesis [8,16]. Although *MYC* is one of the most activated oncogenes implicated in the pathogenesis of human cancers, its activation alone generally cannot induce tumorigenesis; rather, it results in the activation of checkpoints, including those through *p53*, *ARF*, *BIM* and *PTEN* which can cause cell growth arrest or death [17,18,19]. Thus, *MYC* cooperates with many other oncogenic or tumor suppressor genes to initiate tumorigenesis [3]. Its activation is also generally essential for tumorigenesis as shown in several animal models of cancer in which *MYC* alteration is required for tumor initiation, progression or maintenance [19,20,21,22,23]. Therefore, tumors with dysregulated *MYC* have been considered as “MYC-driven” and/or “MYC-addicted” tumors.

A recent report analyzing somatic copy-number alterations (SCNAs) across human cancers has identified 76 amplification regions that are altered at a significant frequency across multiple cancer types, and the most frequent of these focal SCNAs is *MYC* amplifications [24]. The *MYC* oncogene is a central driver in multiple cancers, such as breast cancer [25], liver cancer [26], colorectal carcinoma [27], prostatic neoplasia [28], ovarian cancer and lung cancer [8]. Moreover, high levels of *MYC* deregulation are associated with aggressive conditions and a poor prognosis. For example, in the triple-negative form of breast cancer (TNBC), the most aggressive subtype of breast cancer and the most difficult to treat, *MYC* is amplified in approximately 57% of cases in contrast to only 7–13% in luminal A-type (ER/PR-positive) cancers (a breast cancer subtype with a more favorable outcome) [23,29].

## 4. Mechanisms of MYC Activation/Phosphorylation

In physiologic conditions, *MYC* is under extraordinarily tight regulation by cells [30,31,32,33]. Different factors act to control MYC mRNA expression, stability, export and translation [30,31,32,33]. In addition to transcriptional and mRNA regulation, MYC protein stability and activity are regulated by several post-translational modifications as well as multiple ubiquitin ligases [31,32,33]. The conserved MYC Box 1 (MB1) region of MYC’s transactivation domain is influenced by two sequential and interdependent phosphorylation events on Ser62 (pS62) and Thr58 (pT58). The phosphorylation of MYC enhances its recruitment to target genes [34,35]. MYC is stabilized when it is phosphorylated on Ser62 by extracellular receptor kinase (ERK) or cyclin-dependent protein kinase 2 (CDK2), but it is targeted for degradation when it is phosphorylated on Thr58 by glycogen synthase kinase (GSK-3) via the ubiquitin–proteasome pathway [14,34,35].

The best characterized arm of MYC-induced tumorigeneses relies on the RAS pathway [14]. MYC is activated and stabilized downstream of RAS-induced growth stimuli, which phosphorylate MYC at Ser62. There are at least two effector pathways through which RAS promotes the stability of MYC: the RAF–MEK–ERK kinase cascade and the phosphatidylinositol 3-kinase (PI3K)–protein kinase B (AKT) pathway which inhibits GSK-3β [14,15]. The phosphorylation of MYC on Ser62, after a growth stimulatory signal, results in its stabilization, but also its subsequent phosphorylation at Thr58 by the GSK3 kinase. The phosphorylation of Thr58 then facilitates the dephosphorylation of Ser62 by the protein phosphatase 2A (PP2A), leading to the degradation of MYC through the ubiquitin pathway [36,37]. PI3K–AKT pathway activation by RAS leads to the phosphorylation and inhibition of GSK-3β, facilitating the stabilization of MYC [38]. In contrast, PP2A, which acts as a negative regulator of the PI3K–AKT pathway [39], directly dephosphorylates Ser62 and stimulates the degradation of MYC [15].

PP2A is a major serine/threonine phosphatase with specificity for its substrates. A scaffolding A component, a catalytic C subunit and a third, highly changeable regulatory B subunit make up the heterotrimeric phosphatase PP2A [40]. Structural A and catalytic C subunits have two isoforms, α and β. More than 23 isoforms of the regulatory B subunit exist, and they are divided into four distinct families called B/B55, B′/B56, B″ and B‴. The B56α subunit is the only known B subunit capable of directly inhibiting the stability and activity of MYC [40,41,42]. PP2A-B56α dephosphorylates the Ser62 residue, targeting the phosphorylated MYC protein at Thr58 for ubiquitin-mediated proteasomal degradation [43]. Additionally, PP2A containing the B56α subunit can activate GSK-3β by dephosphorylating it [44]. Conversely, PP2A-B55α can be targeted to MYC in a complex with EYA3 to dephosphorylate Thr58, and this is associated with increased MYC stabiility [42]. Together, increasing our knowledge of key post-translational regulatory events of MYC may provide therapeutic approaches aimed at destabilizing MYC protein [6,45,46].

## 5. The Interplay between RAS and MYC

The intricate interplay between the oncogenes RAS and MYC has been a subject of intense investigation since the groundbreaking discovery in 1983 by Land and colleagues, which unveiled the concept of oncogenic cooperation between these two entities [47]. This discovery revealed the strong reliance that exists among individual oncogenic mutations. Over the past three decades, researchers have sought to unravel the complex mechanisms that underlie the cooperative effects of RAS and MYC in cancer, yet many aspects of this interaction remain shrouded in mystery.

Early studies primarily focused on the individual cell-intrinsic outcomes driven by RAS and MYC. These studies highlighted RAS and MYC synergistic induction and the stabilization of key cell cycle proteins, which played pivotal roles in cellular proliferation and progression [48,49,50], with even MYC stabilization being recognized as a part of this intricate interplay [14]. Studies also delved into the mutual disruption of RAS-induced senescence by MYC [51,52] and the RAS-mediated inhibition of MYC-induced apoptosis [51,53]. Furthermore, researchers delved into the capacity of MYC to overcome barriers to self-renewal in cells driven by RAS mutations [54].

Conversely, critical insights into RAS and MYC’s cooperation emerged from co-transgenic in vivo experiments conducted in mice. These studies unveiled that the synergistic oncogenic partnership between RAS and MYC extends beyond their isolated effects on cells and involves intricate interactions within the complex tumor microenvironment [55,56,57,58]. Such interactions necessitate a comprehensive examination of the dynamic interplay that these oncogenes establish with the tumor stroma. Recent research has taken a focused approach to investigate the collaborative impact of Myc dysregulation on the development and advancement of KRas-driven lung tumors within an in vivo context [55]. This study adeptly delineates the sequential events through which Myc orchestrates the transformation of adenomas into aggressive, inflammatory and immune-suppressed adenocarcinomas. Collectively, these findings offer deeper insights into the complex role of MYC in the progression of tumors. 

To comprehensively understand the full scope of RAS–MYC cooperation, future research endeavors should focus on unraveling the complex network of interactions within the tumor microenvironment and how these interactions influence cancer progression. By delving into the intricate details of this interplay, we may uncover novel therapeutic avenues that target the RAS–MYC axis, potentially leading to transformative strategies for cancer treatment.

## 6. Cell Intrinsic Role of MYC in Tumorigenesis

The precise mechanisms by which the deregulation of the MYC oncoprotein contributes to cancer formation, maintenance and progression are still unclear. MYC deregulation likely induces tumorigenesis via multiple mechanisms, mostly related to its broad ability to regulate the expression of a large number of different genes [1,2,59]. In general, we can state that the cell-intrinsic mechanisms by which MYC induces tumorigenesis include enhancing two fundamental cellular functions: proliferation and survival. MYC enhances cell proliferation via stimulating metabolism, protein synthesis, cell cycle progression and DNA replication; however, this can lead to genomic instability [59]. Then, to ensure cell survival against this stress and for the maintenance of genomic integrity during DNA replication, MYC enhances cell survival via stimulating DNA repair and suppressing cell death [60,61,62]. Taken together, data suggest that MYC plays dual roles in inducing and surviving replication stress.

### 6.1. The Impact of MYC Overexpression on Replication Stress, Genomic Instability and Oncogenic Transformation

MYC overexpression activates downstream genes, stimulating the cell cycle and DNA synthesis and causing genomic instability [59,63,64]. It directly triggers cell cycle progression by activating cyclin D, CDK4 and E2F transcription factors [65,66,67]. MYC affects gene stability, microRNAs and noncoding RNAs, leading to chromosomal alterations [59,68,69,70,71]. *MYC* amplification is associated with various chromosomal changes, including breaks, translocations, deletions, inversions, aneuploidy and extrachromosomal elements [59,70,71].

MYC increases metabolism to fuel tumorigenesis, promoting ATP production and cellular building blocks for cell division and DNA replication, providing a transformation advantage [72,73]. MYC-overexpressing cells exhibit increased glucose and glutamine utilization, stimulating fatty acid and cholesterol synthesis [74,75,76]. This metabolic increase helps sustain high rates of DNA replication in MYC-transformed cells, but the deregulated replication and loss of checkpoints lead to genomic instability and double-stranded DNA breaks (DSBs) [72].

**Figure 1:**
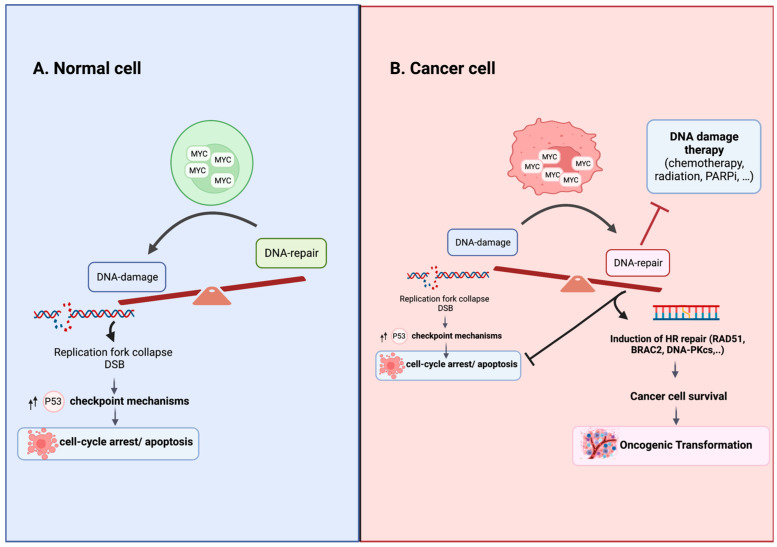
Consequences of MYC Overexpression in Normal and Cancer Cells. (**A**) In normal cells with intact cell cycle checkpoints, MYC overexpression tips the balance in favor of damage over repair by driving replication stress and stimulating checkpoint mechanisms that lead to cell cycle arrest, senescence or apoptosis. (**B**) In cancer cells with altered cellular checkpoints, MYC overexpression upregulates homologous recombination DNA repair, promoting cell survival and resistance to DNA damage agent treatment. The specific mechanisms responsible for oncogenically activating MYC and modulating its functional output in cancer cells are not fully elucidated. The altered post-translational modifications, shifts in binding partners and the formation of multimeric structures are believed to play roles in regulating MYC’s function in cancer cells [81,82,83,84]. Figure was created with BioRender.com, accessed on 2 August 2023.

Thus, deregulated MYC induces DSBs by increasing replication and causing replication stress and the accumulation of reactive oxygen species (ROS) [77,78,79]. MYC-overexpression-induced DSBs in normal cells upregulate the formation of γH2AX foci, a biomarker of DSBs, stimulating cellular checkpoint mechanisms such as cell cycle arrest, apoptosis and premature senescence as part of the DNA damage response (DDR), delaying tumorigenesis [73,80]. MYC overexpression in normal cells can have no impact or varying effects, including proliferative arrest, senescence and apoptosis [17]. However, in MYC-transformed cells, activated MYC promotes DSB repair in response to DNA damage, enabling cancer cell survival [60,61] (Figure 1). The specific mechanisms that oncogenically activate MYC and alter its functional output in cancer cells remain poorly understood. Evidence suggests that modified post-translational modifications, changes in binding partners and multimerization play important roles in this context [34,81,82,83,84,85,86]. Recent studies have demonstrated that MYC forms multimeric structures in response to perturbations, affecting its interactome and enabling tumor cells to proliferate under stressful conditions, limiting DNA double-strand break formation during S-phase [84]. Further research is needed to unravel these intricate molecular mechanisms to advance our understanding of MYC-driven tumorigenesis and identify potential therapeutic targets.

### 6.2. MYC-Induced DSB Repair in Chemoresistance

The accumulation of genomic instability renders the cell genome vulnerable to DSB-induced lethality. The p53 tumor suppressor, also known as the “guardian of the genome”, accumulates in response to DSBs and subsequently induces the transcription of its downstream target genes, which are required for the induction of senescence or apoptosis [77,87]. However, MYC-driven cancer cells survive the accumulation of DNA-damage and maintain DNA replication. In normal fibroblasts, MYC activates the ARF–Mdm2–p53 tumor suppressor pathway, enhancing p53-dependent apoptosis (Figure 1). In contrast, the overexpression of MYC in cancer broadly represses anticancer proteins that promote apoptosis (Figure 1). It has been demonstrated that MYC transgenic mice with MYC-driven tumors also overexpress the anti-apoptosis protein Mdm2, which acts as a negative regulator of p53 [88]. Additionally, MYC has been shown to protect cancer cells from radiation-induced DNA damage and apoptosis, while inducing DNA repair in response to radiation [89]. Thus, it is not surprising that the MYC oncoprotein is closely linked to chemoresistance in different tumor types [60,90,91,92]. It promotes cell survival by increasing DNA repair machinery and suppressing pro-apoptotic processes [60,90,91,92].

Consistent with this, MYC was found to be associated with the promoter region of various DSB repair-related genes, such as NBS1, Ku70, Rad51, BRCA2, Rad50 and the DNA-dependent protein kinase catalytic subunit (DNA-PKcs), and activates their transcription [60,93]. DSBs are repaired through two pathways: homologous recombination (HR) and nonhomologous end joining (NHEJ), which differ in terms of their fidelity and template requirements [77]. HR is predominant during S- and G2-phases, relying on RAD51, RAD51B, RAD51C, RAD51D, XRCC2, XRCC3, BRCA1 and BRCA2 for repair using the sister chromatid as a template [77]. NHEJ functions throughout the cell cycle and depends on the DNA-protein kinase complex and Ku for DSB repair [77]. Silencing MYC in HeLa cells significantly reduces DSB’s repair capability, and MYC regulates RAD51 expression, with MYC induction leading to upregulation and MYC knockdown, resulting in decreased RAD51 expression [60,61,93,94]. MYC’s ability to protect cancer cell genomes from DNA damage can prevent catastrophic damage and promote survival in the face of genomic instability, which can support oncogenic transformation (Figure 1). Targeting MYC may enhance cancer cell sensitivity to DNA damage and serve as a potential strategy for anticancer therapy.

**Figure 2:**
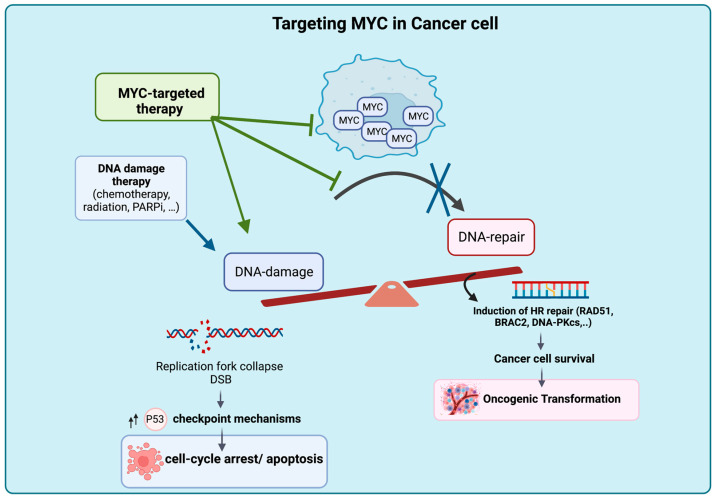
Proposed Mechanism of Tumor Intrinsic Cell Death Upon MYC Deactivation. Targeting MYC results in accumulation of DNA damage due to the blockage of DNA DSB repair, leading to cell death and rendering the cancer cell genome vulnerable to the effects of DNA-damaging agents. Figure was created with BioRender.com, accessed on 2 August 2023.

The suppression of MYC expression or function can reverse tumorigenesis by reversing abnormal DNA replication and DNA repair processes, leading to cell cycle arrest, apoptosis, senescence and the accumulation of DNA damage [22,60,90,91,92,95]. Targeting MYC in cancer cells with high genomic instability can induce cell death and enhance vulnerability to DNA-damaging agents (Figure 2). The inhibition of MYC-mediated DSB repair following treatment with DNA-damaging agents holds promise for overcoming MYC-induced drug resistance and chemoresistance.

## 7. MYC as a Regulator of the Tumor Microenvironment Leading to Drug Resistance

**Figure 3:**
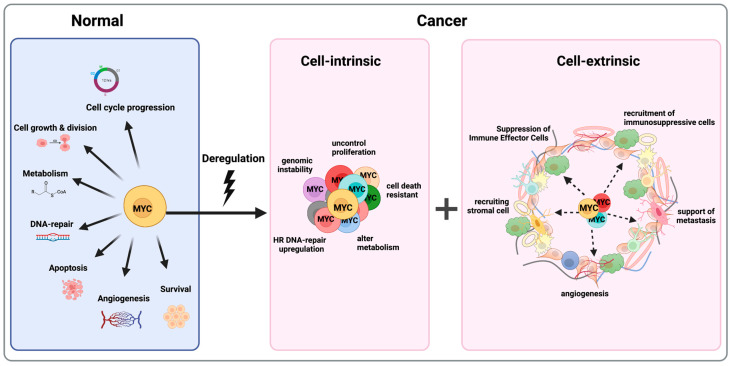
MYC is a Master Regulator of Almost All the Hallmarks of Cancer. MYC controls a variety of cellular functions (on the **left**); deregulation of MYC leads to tumors “addicted” to MYC because of both tumor cell-intrinsic (in the **middle**) and cell-extrinsic mechanisms (on the **right**). Figure was created with BioRender.com, accessed on 2 August 2023.

Tumorigenesis is a complex process that not only affects malignant cells’ genetic events but also influences the surrounding tumor microenvironment (TME). The TME includes endothelial cells, fibroblasts and immune cells among many others, in addition to the extracellular matrix produced by these cells. The crosstalk between tumor cells and mesenchymal stromal cells is thought to be critical for both cancer development and drug resistance [96,97]. More recently, novel studies have shown that MYC plays a role in tumorigenesis in cell intrinsic signaling and has a broader spectrum of functions in the tumor microenvironment [55,98,99,100] (Figure 3). It is acknowledged that stromal cells alter tumor cell drug responses, with cancer-associated fibroblasts (CAFs) and inflammatory cells comprising most stromal cells in the tumor microenvironment. Variations in fibroblast cells and immune profiles have been linked to tumorigenesis and therapeutic responses [101,102].

Apart from its cell-intrinsic role in tumorigenesis, MYC plays a crucial role in shaping the tumor microenvironment and establishing a nurturing niche for cancer cells (Figure 3). Through its transcriptional activity, MYC orchestrates a variety of molecular changes that contribute to the development of a tumor-permissive microenvironment and educating tumor-infiltrating cells [103,104]. This microenvironment not only promotes cancer cell survival and growth but also fosters drug resistance. MYC contributes to angiogenesis, CAFs’ metabolic changes, immune evasion, invasion and migration, which all lead to distant drug resistance [55,105,106,107].

One key aspect of MYC’s programming of the tumor microenvironment is its ability to stimulate angiogenesis, the formation of new blood vessels. MYC promotes the secretion of angiogenic factors such as vascular endothelial growth factor (VEGF), which stimulates the growth of blood vessels, ensuring an adequate oxygen and nutrient supply to the tumor [98,107,108]. This enhanced vascular network facilitates cancer cell survival and enables the cells’ rapid proliferation. Emerging evidence indicates that MYC upregulation is not limited to cancer cells but also extends to cancer-associated fibroblasts (CAFs) [109]. CAFs have been shown to play a crucial role in promoting tumor angiogenesis [110,111]. CAFs achieve this by secreting cytokines that attract endothelial cells, facilitating the formation of tumor-associated blood vessels and nodal metastases. This finding aligns with previous research by Baudino et al., who demonstrated in mouse models that MYC plays a crucial role in regulating cytokines involved in lymphangiogenesis, including VEGF-C and VEGF-D [112]. Consequently, the upregulation of MYC expression may contribute to the development of nodal metastases. The presence of MYC-expressing fibroblasts in the local metastatic environment assists in promoting colonization by creating a microenvironment conducive to lymphangiogenesis, thereby supporting the survival of cancer cells [109,112]. In addition, MYC expression in CAFs directly regulates the expression of genes involved in glucose metabolism, including lactate dehydrogenase A [109,113]. It has been reported that CAFs exhibit an elevated expression of glycolytic enzymes [114]. This implies that cancer cells can take advantage of altered CAF metabolism to facilitate tumor growth and vascularization [109,113,114]. These observations strongly suggest that MYC plays an essential role in regulating CAFs within the tumor microenvironment, further emphasizing its multifaceted involvement in cancer progression and the shaping of the tumor microenvironment.

### 7.1. Immune Evasion and MYC

MYC promotes immunosuppression and contributes to the recruitment of immunosuppressive cells within the tumor microenvironment [55,115]. This section discusses the mechanisms by which MYC supports immune evasion in cancer.

#### 7.1.1. MYC Induces the Recruitment of Immunosuppressive Cells 

MYC plays a significant role in promoting the recruitment of immunosuppressive cells within the tumor microenvironment, including regulatory T cells (Treg) [23,55,103,116,117]. MYC in tumor cells influences Treg accumulation, activation and metabolic programming [118]. MYC overactivation promotes glycolysis in tumor cells, creating a low-glucose environment that favors Treg generation [119,120,121]. MYC also regulates the secretion of chemokines and cytokines, such as CCL22, CCL17, TGF-β and IL-10, which attract and activate Tregs [55,107,122]. Additionally, MYC influences the recruitment of myeloid-derived suppressor cells (MDSCs) and tumor-associated macrophages (TAMs) [103,117,123,124,125,126,127,128]. These immunosuppressive cells establish an immune-evading microenvironment that hampers effector immune cell function and promotes tumor growth.

#### 7.1.2. Suppression of Immune Effector Cells and Escape from Immune Recognition

Effector immune cells, including T cells and NK cells, play a crucial role in recognizing and eliminating cancer cells. MYC can modulate the function of these cells to suppress their cytotoxic activity [55,129]. For instance, MYC activation in cancer cells can downregulate the expression of major histocompatibility complex (MHC) molecules, impairing antigen presentation and the subsequent activation of T cells [129]. Additionally, MYC-induced metabolic reprogramming creates a nutrient-deprived microenvironment that impairs multiple immune cell types [55,119,120,121]. Furthermore, MYC contributes to immune evasion in tumor cells by inducing the expression of PD-L1, which suppresses the attack from immune cells against the cancer cells [55,130,131,132]. These mechanisms contribute to the establishment of an immunosuppressive tumor microenvironment, allowing cancer cells to evade immune surveillance, which suggests that targeting MYC could be a therapeutic strategy to counteract immune evasion and enhance cancer treatment outcomes.

MYC inhibition reverses the immunosuppressive tumor microenvironment, restores immune cell activation and enhances the production of immune-stimulatory molecules [133,134,135,136]. Preclinical studies demonstrate the potential of MYC inhibition to improve immune response and synergize with immunotherapies [137,138,139]. Targeting MYC is promising for restoring immune response, modulating the tumor microenvironment and improving cancer patient outcomes.

## 8. MYC as a Therapeutic Target for Cancer

Experimental evidence has demonstrated that inhibiting MYC expression can reverse tumorigenesis, providing a proof of concept for the pharmacological targeting of this oncoprotein to impede tumor cell growth [2,99,140]. However, developing specific pharmacological agents to target MYC is challenging due to its unique characteristics. MYC’s disordered structure, lack of hydrophobic pockets and absence of catalytic activity make it difficult to bind with small molecules or conventional enzyme inhibitors. Moreover, MYC’s nuclear localization presents challenges for targeting with large molecules, such as monoclonal antibodies.

**Figure 4:**
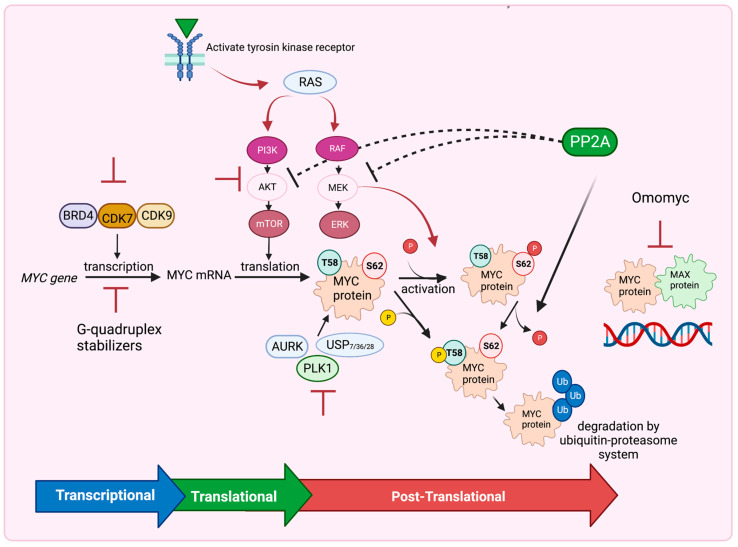
Several Strategies to Target MYC. Inhibitors of BRD4, CDK7 and CDK9 or G-quadruplex stabilizers inhibit MYC expression at the transcriptional level. Inhibition of the PI3K/AKT/mTOR pathway blocks MYC translation, whereas inhibition of RAF/MEK/ERK pathway, USP7, 28, 36, AURK and PLK1 inhibitors destabilize MYC at the posttranslational level. PP2A also dephosphorylates MYC at Ser62 and destabilizes MYC leading to MYC degradation by the ubiquitin–proteasome system. PP2A also inhibits the PI3K/AKT/mTOR and RAF/MEK/ERK pathways. Omomyc drug functions to interrupt the MYC–MAX dimeric complex binding to DNA. Figure was created with BioRender.com, accessed on 5 August 2023 and adapted from [14,141,142].

Despite these obstacles, recent pharmaceutical-based strategies have emerged to target MYC and hinder tumor growth [2,14,46,140,141,142]. These approaches include inhibiting MYC expression at the transcriptional level, blocking MYC translation through the PI3K/AKT/mTOR pathway, destabilizing MYC using inhibitors of USP7, AURKA, and PLK1 or activators of PP2A at the posttranslational level, and utilizing Omomyc to disrupt the MYC/MAX dimeric complex binding to DNA. These strategies offer potential avenues to inhibit MYC’s function and suppress tumor growth (Figure 4). Further research and development in this field are essential for advancing our understanding and therapeutic targeting of MYC in cancer treatment.

### 8.1. Targeting MYC Gene Transcription

Targeting *MYC* transcriptional regulation is a promising strategy for cancer treatment [46,140,143]. The BRD4 inhibitor (JQ1) competes with BRD4 for binding to acetylated lysines, displacing BRD4 from super-enhancers within the *MYC* oncogene and reducing MYC expression, resulting in anti-cancer effects in hematopoietic cancers, PDAC and MYCN-driven cancers [144,145]. The inhibition of CDK7 and/or CDK9, which is critical for *MYC* transcription, reduces MYC expression and downregulates MYC target genes. Inhibitors against CDK7 and CDK9 demonstrate potent anti-tumor effects in MYC-driven cancers, including T cell acute lymphoblastic leukemia, mixed-lineage leukemia, neuroblastomas and small cell lung cancers [146]. Additionally, the *MYC* promoter possesses a structurally actionable element [143,147]. Several studies have demonstrated the potential of specific small-molecule ligands, such as cationic porphyrins and quindolines (e.g., CX-33543 or quarfloxin), to stabilize G-quadruplexes within the *MYC* promoter, leading to the downregulation of MYC expression [143,148,149]. Further research should focus on optimizing these therapies and exploring combination treatments for enhanced efficacy.

### 8.2. Targeting MYC mRNA Translation

Targeting MYC mRNA translation provides an alternative approach to combating MYC-driven cancers [143,150,151]. The PI3K/AKT/mTOR pathway, frequently dysregulated in various cancers, plays a crucial role in protein synthesis through mTOR complexes 1 and 2 (mTORC1 and mTORC2). The mTORC1-mediated phosphorylation of eukaryotic translation initiation factor 4E-binding protein 1 (4EBP1) releases its inhibition on eIF4E, leading to the enhanced translation of mRNAs with long 5′-untranslated regions (5′-UTRs) and complex RNA secondary structures, including MYC mRNA. The pharmacological inhibition of the PI3K/AKT/mTOR pathway significantly reduces MYC levels and demonstrates therapeutic efficacy in MYC-driven cancers [150,151]. Additionally, cytoplasmic polyadenylation element-binding protein (CPEB) controls polyadenylation-induced translation and can recognize cytoplasmic polyadenylation elements (CPEs) in the 3-UTRs of MYC mRNA. CPEB inhibits c-MYC expression by promoting the deadenylation and decay of its mRNA [152]. CPEB family proteins are often downregulated in human cancers, so restoring their expression could lead to MYC inhibition in MYC-driven cancers [153].

### 8.3. Targeting MYC Stability

Targeting MYC stability offers a potential approach for suppressing MYC-dependent cancers [32,33]. The ubiquitin–proteasome system tightly regulates MYC stability, with phosphorylation at Thr58 triggering polyubiquitination by the E3 ligase FBW7 and subsequent proteasomal degradation [154,155]. Deubiquitinating enzymes, such as USP28, USP36 and USP7, counteract MYC degradation mediated by FBW7, leading to MYC stabilization and tumor cell proliferation [156,157]. Additionally, AURKA and PLK1 play crucial roles in maintaining MYC expression. AURKA forms a complex with N-MYC, protecting it from FBW7-mediated degradation, and inhibitors of AURKA disrupt the MYC–AURKA complex, promoting N-MYC degradation and tumor regression [158]. PLK1 and MYC create a positive feedforward activation loop that sustains a high expression, and PLK1 inhibitors induce apoptosis in MYC-overexpressing tumor cells, highlighting their potential as therapeutics for MYC-dependent cancers [159]. Targeting these mechanisms could destabilize MYC and offer therapeutic benefits for MYC-driven cancers.

The PP2A serine/threonine phosphatase is frequently inhibited in most human cancers, and it primarily functions as a tumor suppressor by diminishing the activation of key oncogenic regulators, including MYC, ERK and AKT [160]. PP2A directly dephosphorylates MYC, resulting in its degradation, making MYC one of the well-characterized substrates of PP2A [160,161,162].

Recent studies have demonstrated the effectiveness of re-engineering FDA-approved tricyclic neuroleptics, particularly small-molecule activators of PP2A (SMAPs), in inducing apoptosis, promoting the dephosphorylation of PP2A targets such as MYC and AKT and suppressing tumor growth in mouse models [20,163].

A recent study by Leonard et al. provides insight into the direct activation of PP2A by an SMAP molecule called DT-061, one of the lead-engineered compounds based on tricyclic neuroleptics [164]. The researchers describe the 3D structure of the DT-061-bound PP2A trimeric complex and reveal that DT-061 occupies a unique intersubunit pocket, which directly binds and selectively stabilizes the PP2A-B56α holoenzyme [164]. This binding and stabilization mechanism of DT-061 on PP2A-B56α enhances its antitumor function [164]. The dephosphorylation of the most-studied MYC-activating phosphorylation event on Serine 62 by PP2A-B56α suggests that DT-061 has potential as an approach for targeting active MYC through the stabilization of PP2A-B56α [161,164]. The in vivo efficacy of DT-061 in murine tumor models further supports its therapeutic potential, although further investigation is needed to assess its effects on immune function in disease [20].

### 8.4. Targeting the MYC–MAX Complex

The formation of the MYC/MAX complex is essential for MYC to bind to DNA and activate the transcription of target genes [165]. This complex adopts a parallel, left-handed, four-helix bundle structure, in which each monomer consists of two R-helices separated by a loop [166]. Although this structure does not exhibit obvious binding sites for small-molecule inhibitors, researchers have conducted screenings to identify molecules that can block the interaction. Among them, the peptide mimetic IIA6B17 has been identified as a small-molecule inhibitor of MYC/MAX dimerization [167]. Another compound known as 10058-F4 has demonstrated the ability to disrupt the MYC/MAX complex specifically in HL60 cells [166]. Omomyc, a well-known inhibitor, is a mutant mini-peptide with a basic helix–loop–helix structure that sequesters MYC in an inactive complex, preventing MYC-induced tumorigenesis in multiple mouse tumor models [168].

### 8.5. Enhancing Therapeutic Efficacy: Combining MYC Targeting with DNA Damage Agents, including PARP Inhibitors

PARP inhibitors (PARPi) are a novel class of anticancer therapies that competitively bind to the catalytically active site of PARP molecules, interfering with their DNA repair function by competing with NAD+ [169]. The PARP inhibitors exhibit varying degrees of potency in terms of enzymatic inhibition and PARP trapping effects [170,171,172]. For example, olaparib and talazoparib have similar levels of catalytic inhibition, but talazoparib shows approximately a 100-fold higher potency than olaparib in trapping PARP–DNA complexes [173,174]; however, the clinical significance of these modes of action are still under study. These inhibitors have shown effectiveness in treating tumors with defects in homologous recombination repair (HR). Specifically, PARP inhibitors have been utilized to target tumors harboring mutations in the key HR genes, Breast Cancer Associated 1 and 2 (BRCA1 and BRCA2) [175]. Several PARP inhibitors have received approval for the treatment of BRCA-mutated ovarian, breast and pancreatic cancers. There are currently 269 clinical trials registered to investigate the potential of PARP inhibitors as an anticancer therapy in chemo-resistant germline or somatic BRCA1/2 mutated breast, ovarian, lung and pancreatic cancers [169].

Acquired resistance to PARPi is a significant challenge in cancer treatment, and one of the mechanisms behind this resistance is the restoration of homologous recombination (HR) capacity [175]. This can occur through reversion mutations, in which BRCA1/2 function is restored by secondary mutations [176,177]. Studies have shown that reversion mutations in BRCA1/2 are observed in patients with breast, ovarian and pancreatic carcinomas and are associated with the development of PARPi-resistant tumors [176,177,178]. Additionally, high levels of MYC have been found to enhance the expression of RAD51, a key protein involved in homologous recombination repair (HRR) [93,94]. Elevated levels of c-MYC and RAD51 in TNBC patients have been associated with resistance to PARP inhibitors [179,180].

The prevalence of BRCA1/2 mutations among patient cases is relatively low, and a substantial number of these patients develop resistance to PARP inhibitors, resulting in treatment failure [181,182,183]. Consequently, there is a considerable focus on exploring combination therapies to enhance the effectiveness of treatments and broaden the scope of patients who can derive benefits from PARP inhibitors. One approach is to combine PARPi with agents that induce HR defects in tumors with intact HR function, thereby rendering them sensitive to PARP inhibition [184]. Additionally, strategies that block DNA repair pathways by inducing hypoxia or interfering with DNA damage cell cycle checkpoints have been explored to enhance the effectiveness of PARPi. Examples include inhibitors targeting signaling through the PI3K pathway and cell cycle checkpoints [184]. The upregulation of MYC has been linked to the activation of the homologous recombination DNA repair pathway, including the increased expression of RAD51 [60,61,93,94,185]. Indeed, it has been demonstrated that these molecular alterations, specifically upregulated RAD51 expression, contribute to the development of resistance to PARP inhibitors in cells with defective BRCA1 [179]. Recent evidence has shown that inhibiting the downstream signaling of MYC through cyclin-dependent kinase (CDK) inhibitors can enhance cancer cell sensitivity to PARP inhibitors, regardless of the BRCA status, in triple-negative breast cancer (TNBC) [180]. Despite these observations, the specific role of MYC in regulating DNA repair mechanisms and its impact on therapy response have often been overlooked. This intriguing association between MYC overexpression, enhanced HR DNA repair and resistance to DNA-damaging agents, such as PARPi, has prompted the need for further investigation.

## 9. Discussion and Future Directions

In conclusion, MYC is a multifunctional nuclear phosphoprotein that plays a critical role in regulating various cellular processes, including cell growth, the cell cycle, differentiation, metabolism and DNA repair. MYC alterations are prevalent in human cancers, contributing to tumorigenesis and drug resistance. Even though oncogenic MYC leads to the production of rapidly dividing cancer cells and an increase in genomic instability, one would expect these cells to be more vulnerable to DNA-damaging therapy. However, the opposite happens; high-MYC cancer cells exhibit prolonged survival even after DNA-damaging chemotherapy [21,23,186]. This evidence strongly indicates that oncogenic MYC in cancer cells triggers the development of a highly efficient DNA repair system, effectively countering the genomic damage induced by rapid proliferation, as well as chemotherapy and radiation therapy [89]. Understanding MYC-induced DNA repair mechanisms may offer opportunities to enhance cancer cell sensitivity to DNA-damaging agents and overcome drug resistance.

Moreover, MYC’s impact extends beyond cell-intrinsic signaling, influencing the tumor microenvironment by promoting angiogenesis, immune evasion and the recruitment of immunosuppressive cells. Targeting MYC in cancer cells and the tumor microenvironment may provide a promising strategy for anticancer therapy and improving treatment outcomes. Despite the challenges in targeting MYC directly, emerging pharmaceutical-based approaches offer hope for inhibiting MYC expression and function as well as impeding tumor growth. Numerous studies have consistently demonstrated that targeting MYC activity or expression results in a notable reduction in tumor growth across diverse preclinical tumor models [20,21,46,187,188]. Notably, emerging evidence highlights the impact of MYC deregulation in suppressing the immune response to tumors, whereas inhibiting MYC activity shows promise in stimulating an anti-tumor response [55,99,100,133,134,135]. Future research should focus on establishing the broader potential of MYC inhibitors in enhancing anti-tumor immunity. Continued research in this area is vital for advancing our understanding of MYC’s role in cancer and developing effective therapeutic interventions targeting this master oncoprotein.

Future directions in MYC-related research encompass various aspects aimed at deepening our understanding of its role in cancer and harnessing its potential for therapeutic applications:

1. Unraveling DNA Repair Mechanisms: Investigating the precise mechanisms underlying MYC-induced DNA repair can unveil novel vulnerabilities in cancer cells. This knowledge could lead to the development of strategies that sensitize high-MYC cancer cells to DNA-damaging agents, ultimately overcoming drug resistance.
2. Microenvironment Modulation: Further exploring how MYC impacts the tumor microenvironment, especially its influence on immune evasion and angiogenesis, can provide insights for designing therapies that not only target cancer cells but also disrupt the supportive network around them. This could potentially enhance the effectiveness of anticancer treatments.
3. Refining MYC Inhibitors: Despite challenges, refining pharmaceutical-based approaches to inhibit MYC expression and function remains a promising avenue. Future research could focus on designing more potent and selective MYC inhibitors that effectively halt its oncogenic effects, leading to improved outcomes in cancer treatment.
4. Immunomodulation Strategies: Understanding the interplay between MYC deregulation, immune suppression and anti-tumor immunity is critical. Exploring the potential of MYC inhibitors to enhance anti-tumor immune responses could open up new avenues for immunomodulatory therapies.
5. Patient-Derived Models: Utilizing patient-derived models, such as organoids and xenografts, can offer more clinically relevant insights into MYC-targeted therapies and help bridge the gap between laboratory research and clinical application.
6. Clinical Translations: Transitioning findings from preclinical models to clinical settings is vital. The rigorous testing of MYC inhibitors in clinical trials across different cancer types can help us evaluate their safety, efficacy and potential to improve patient outcomes.
7. Combination Therapies: Exploring combination therapies that integrate MYC inhibition with existing treatments, such as DNA-damaging agents or immunotherapies, might offer synergistic effects and enhance therapeutic responses. Identifying optimal combinations is a promising avenue for future investigations.

In summary, future research endeavors should focus on gaining a deeper understanding of MYC’s multifaceted roles in cancer biology and translating these insights into innovative therapeutic strategies. This includes refining existing approaches, exploring combinatorial treatments and uncovering novel facets of MYC’s influence on tumor progression and the immune response. Such efforts are promising for advancing cancer treatment and improving patient outcomes.
